# Prognostic significance of synergistic hexokinase-2 and beta2-adrenergic receptor expression in human hepatocelluar carcinoma after curative resection

**DOI:** 10.1186/s12876-016-0474-8

**Published:** 2016-06-03

**Authors:** Zhi-Feng Zhang, Xiao-Sha Feng, He Chen, Zhi-Jun Duan, Li-Xia Wang, Dong Yang, Pi-Xu Liu, Qiu-Ping Zhang, Yan-Ling Jin, Zhi-Gang Sun, Han Liu

**Affiliations:** Department of Gastroenterology, The First Affiliated Hospital of Dalian Medical University, 116000 Dalian, Liaoning Province China; Institute of Cancer Stem Cell, Dalian Medical University, 116000 Dalian, Liaoning Province China; Department of Pathology, The First Affiliated Hospital of Dalian Medical University, 116000 Dalian, Liaoning Province China

**Keywords:** Hepatocellular carcinoma, Hexokinase-2, Beta2-adrenergic receptor, Prognosis, Immunohistochemistry

## Abstract

**Background:**

Hexokinase-2 (HK2) and Beta2-adrenergic receptor (Beta2AR) are overexpressed in hepatocellular carcinoma (HCC) tissues and associated with poor prognosis. However, the synergistic effect of HK2 and Beta2AR in HCC prognosis is not elucidated. The present study aims to investigate the association between HK2 and Beta2AR expressions in HCC tissues, and to evaluate the synergistic effect of HK2 and Beta2AR in HCC prognosis.

**Methods:**

Immunohistochemistry of HK2 and Beta2AR was performed on 155 paraffin embedded HCC samples retrieved from the archives of pathology department. Corresponding clinical data and prognostic data were collected through searching medical record systems, death registration systems and interviews with patient families. Spearman correlation test was performed to evaluate the association between HK2 and Beta2AR expression. Kaplan-Meier survival curves and Cox regressions were employed to evaluate HK2 and Beta2AR expression in HCC prognosis, respectively and synergistically.

**Results:**

109 of 155 HCC patients reached the death point, the survival time of HCC patients was 46.23 ± 31.01 months after curative surgical resections of HCC. Kaplan-Meier survival analysis showed that large tumor size (more than 5 cm) (hazard ratio (HR) = 8.42, 95 % confidence interval (CI) = 3.81–18.59, *P* < 0.0001), advanced TNM stage (III and IV stages) (HR = 2.09, 95%CI = 1.21–3.62, *P* < 0.001) and AFP more than 20 μg/L (HR = 1.49, 95%CI = 1.02–2.18, *P* = 0.0302) were predictors for poor prognosis. HK2 and Beta2AR positive expression was detected in 66 (42.58) and 122 (78.71 %) HCC samples respectively. In univariate analysis, HK2(+) (HR = 2.70, 95%CI = 1.76–4.15, *P* < 0.0001) and Beta2AR(+) (HR = 4.61, 96%CI = 3.14–6.76, *P* < 0.0001) were associated with poor prognosis. In multivariate analysis, HK2(+) (*P* < 0.0001) and Beta2AR(+) (*P* < 0.0001) were also associated with poor prognosis. HK2(+)/Beta2AR(+) in HCC samples had poorer prognosis compared with HK2(−)/Beta2AR(−) in both univariate analysis (HR = 4.69, 95%CI = 2.91–7.57, *P* < 0.0001) and multivariate analysis (*P* < 0.0001). HK2(+)/Beta2AR(+) in HCC samples had poorer prognosis compared with HK2(−)/Beta2AR(+) in both univariate analysis (HR = 1.76, 95%CI = 1.17–2.64, *P* = 0.003) and multivariate analysis (*P* = 0.004).

**Conclusion:**

HK2 and Beta2AR play important roles in HCC progression. HK2 and Beta2AR expression in HCC is correlated positively. Beta2AR may increase HCC invasion and metastasis in collaboration with HK2. HK2 and Beta2AR can predict HCC prognosis both independently and synergistically.

## Background

Hepatocellular carcinoma (HCC) is the fifth most common cancer in men and seventh most common cancer in women worldwide [1]. HCC is also the third most common cause of death from carcinomas worldwide [1, 2]. Although new treatment modalities for HCC have been developed in recent years, improvement of the five-year survival rates mainly relies on diagnosis at early stages of HCC. Early stage HCC can be treated with curative surgical resections, radiofrequency ablation, percutaneous ethanol injection and transarterial chemoembolization [2]. Moreover, only curative surgical resection has the possibility to cure HCC. As for the advanced stage HCC, although tyrosine kinase inhibitors, such as Sorafenib and Linifanib have been developed to improve survival rates, the cure of advanced stage HCC is rarely achieved [3, 4]. Therefore, how to choose the optimal therapy for the individual patient in order to maximize the survival time is of great importance to both clinicians and patients. Another issue of importance to clinicians and patients is to predict prognosis of HCC especially for the patients underwent curative HCC resections and for the patients with advanced stage HCC. Proper prediction of prognosis can guide clinicians to choose the right therapies and help patients to arrange the remaining life appropriately. To date, no individual biomarker can predict prognosis of HCC accurately. The combination of two or more correlated biomarkers for HCC prognosis may improve the power of prognosis predictions of HCC.

Beta2-adrenergic receptor (Beta2AR) is a transmembrane G protein-coupled receptor (GPCR), which can regulate cell proliferation via cyclic adenosine monophosphate and protein kinase A pathways [5]. Beta2AR are overexpressed in multiple cancers especially in gastrointestinal cancers and HCC [6–8]. It has been proved that Beta2AR agonist can promote DNA synthesis and beta-blockers can block DNA synthesis in pancreatic ductal carcinoma cell lines [7]. Selective Beta2AR blockage can also suppress colorectal cancer growth via EGFR-Akt/ERK signaling, G1-phase arrest and apoptosis [9]. Through increasing DNA synthesis, accelerating cell cycle and decreasing apoptosis, activation of Beta2AR promotes cancer growth, invasion and metastasis. High expression of Beta2AR may play a role in the canceration of injured hepatocytes, and may represent high grade of malignancy of HCC. So high expression of Beta2AR in HCC tissues is proposed be a biomarker for poor prognosis, which is supported by a previous survival study of HCC with immunohistochemistry [10].

Besides increased DNA synthesis, accelerated cell cycle and decreased apoptosis, another feature of cancer is the energy shift to aerobic glycolysis called Warburg effect, even in adequate oxygen environments [11]. Accelerated aerobic glycolysis is of importance to cancer cell survival, growth and metastasis [11]. Hexokinase (HK) is a key enzyme in glycolysis, to date, four HK isoforms were identified in mammals [12]. It is shown that HK2 is overexpressed in HCC tissues [13, 14]. Overexpression of HK2 may also represent high grade of malignancy. Two survival studies proved this hypothesis, and showed that high level expression of HK2 was an independent poor prognostic biomarker for HCC [13, 14].

Moreover, HK2 and Beta2AR are not independent, they are related in HCC development. Recently, one study indicates that activation of Beta2AR can promote HK2 expression, and inhibition of Beta2AR can decrease HK2 expressions in breast cancer cell lines [15]. It is deduced that Beta2AR might increase the grade of malignancy of cancers via promoting HK2 expression. However, the relationship between Beta2AR and HK2 expression in HCC tissues is not fully elucidated. We postulate that Beta2AR and HK2 expression in HCC tissues correlates positively; overexpression of HK2 and beta2AR in HCC can predict prognosis of HCC patients synergistically; Beta2AR may increase the grade of malignancy of HCC via promoting HK2 expression. In the current study, we performed immunohistochemistry assays to prove the above hypotheses.

## Methods

### HCC patients and tissue samples

This study is a retrospective study. 160 HCC patients underwent curative resections between January 2000 and December 2013 in the First affiliated hospital of Dalian Medical University were included in our study. Surgical treatment selection was mainly based on the Barcelona Clinic Liver Cancer (BCLC) algorithm [16]. Moreover, ten HCC patients with regional metastasis underwent HCC resections and lymph node dissections were also included into our study. Paraffin embedded pathologic samples were retrieved from the archives of the Department of Pathology. All the tissue sections were evaluated by experienced pathologist to confirm the diagnosis of HCC. Cholangiocellular carcinoma and other carcinomas of the liver were excluded during pathology evaluation. HCC patients accompanied with other site carcinomas were also excluded from our study. Because Beta2AR and HK2 were associated with metabolism, HCC patients accompanied with metabolic diseases including diabetes mellitus and thyroid diseases were also excluded from our study. This study was approved by Medical Ethics Committee of First Affiliated Hospital of Dalian Medical University. Consent was obtained from all participants (alive HCC patients or relatives of deceased HCC patients).

### Survival data and clinical data collection

Clinical and laboratory data of each included patient during the hospital stay for curative resection were retrieved from the medical record system of the First Affiliated Hospital of Dalian Medical University. The computed tomography (CT) scanning and magnetic resonance images (MRI) of each patient were collected via Picture Archiving and Communication Systems. The records of operational procedures and observations during operations were also collected via medical record system. The CT images, MRI images and observations during operation were used to evaluate the tumor size (maximum diameter of the tumor). The survival data and the death date of each patient were retrieved through telephone interviews with patient families, and confirmed by searching the municipal death registration system of Dalian (the final collection date was 20th March 2014, the data collection period was limited in two weeks). Overall survival was used to evaluate HCC patient survival after surgical curative resections. Paraffin embedded pathologic samples of each patient were reevaluated by experienced pathologist to determine the differentiation grade of HCC according to Edmondson-Steiner histopathologic grading [17]. The HCC staging evaluation before operation was based on American Joint Commission on Cancer 7th edition TNM system [18].

### Immunohistochemistry and density scoring

Immunohistochemistry was performed with the MaxVisionTM AP kit (Fuzhou Maixin Biotech. Co., Ltd, Fuzhou, China) according to the manufacture manuals. HK2 expression was evaluated with a rabbit anti-human HK2 monoclonal antibody (Cell Signaling Technology, Inc., Danvers, MA, USA) in accordance with the manufacturer’s protocol with antibody dilution of 1:50. Beta2AR was evaluated using a rabbit anti-human Beta2AR monoclonal antibody (Abcam, Cambridge, MA, USA) in accordance with the manufacturer’s protocol with antibody dilution of 1:50. Both the intensity and proportion of stained cells in a HCC section (200×) were inspected and evaluated. Staining intensity was classified with a four grade system: no staining (0), weak (1), moderate (2), strong (3). The percentage of positive stained cells was also classified into a four scale system: <5 % (0), 5–25 % (1), 26–50 % (2), >50 % (3). All the scores were based on the mean scores of ten randomly selected fields of tissue sections. The staining intensity score plus the percentage score made the final score of each sample (the final score ranged from 0 to 6). If the final score is more than 3, the sample was determined to be positive. Otherwise, the sample was determined to be negative.

### Statistical analysis

Pearson Chi-square test was used to analyze the associations between protein expression and clinical data. Survival time was shown as mean and standard deviation (SD). One-way ANOVA analysis and least significant difference (LSD) test were employed to determine the difference of live time among different groups. Spearman correlation test was performed to evaluate the association between HK2 and beta2AR expression with the final scores. Kaplan-Meier survival curve was employed to evaluate HK2 expression, Beta2AR expression and clinical data in HCC prognosis, respectively and synergistically. Cox regression analysis was also performed for multivariate analyses (all the selected clinical data was included in Cox regression) for HK2, Beta2AR and clinical data in HCC prognosis. *P* < 0.05 was considered statistically significant. Pearson Chi-square test and One-way ANOVA analysis were performed with the SPSS16.0 statistical software package (SPSS Inc., Chicago, IL, USA). Survival analysis and Cox regression analysis were performed with MedCalc 11.4.2.0 (MedCalc Software bvba, Acacialaan, Ostend, Belgium). Survival curve, Cox regression curve, Spearman correlation diagram and One-way ANOVA diagram were depicted with MedCalc 11.4.2.0 (MedCalc Software bvba, Acacialaan, Ostend, Belgium).

## Results

### The association between clinical data and survival

160 paraffin embedded HCC samples were retrieved from the archives of pathology department. Complete clinical data and prognostic data were collected through searching medical record systems and taking interviews with patient families. The survival data were confirmed by searching municipal death registration system of Dalian. Survival information of 155 patients was retrieved successfully, the rate of lost to follow-up was 3.13 %. 155 patients were included into final analyses. 109 (70.32 %) HCC patients reached the death point, the survival time of HCC patients was 46.23 ± 31.01 months after curative surgical resections of HCC. Kaplan-Meier survival analysis showed that large tumor size (more than 5 cm) (hazard ratio (HR) = 8.42, 95 % confidence interval (CI) = 3.81–18.59, *P* < 0.0001), advanced TNM stage (III and IV stages) (HR = 2.09, 95%CI = 1.21–3.62, *P* < 0.001) and AFP more than 20ug/L (HR = 1.49, 95%CI = 1.02–2.18, *P* = 0.0302) were predictors for poor prognosis. Age, gender, Hepatitis viral infection, tumor number, tumor differentiation grade and post operational treatment are not associated with prognosis of HCC patients. Cox regression showed that only large tumor size and advanced TNM stage were associated with poor prognosis of HCC patients.

### HK2 and Beta2AR are over expressed in HCC

**Fig. 1:**
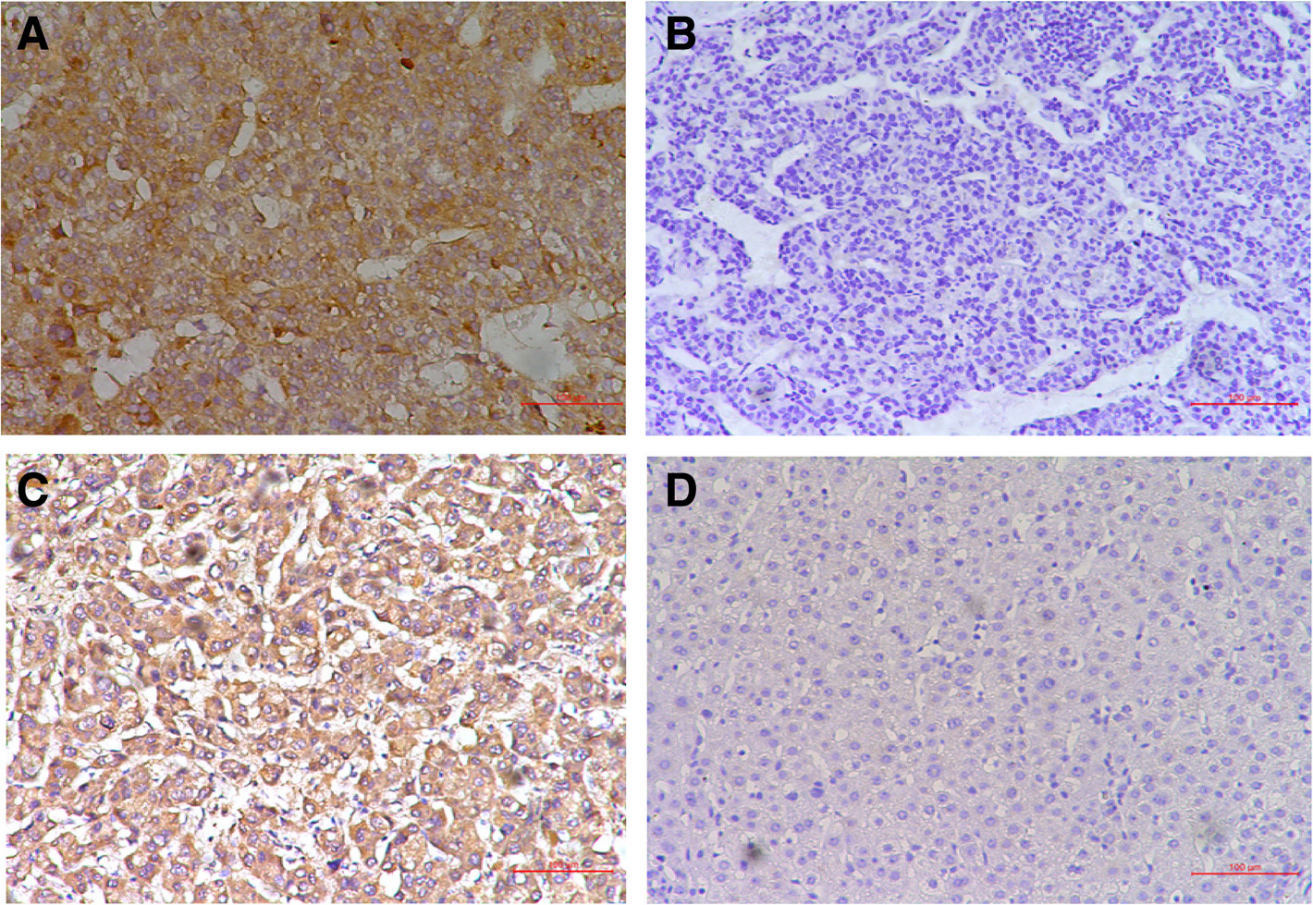
Immunochemistry of HCC tissues. **a** Representative positive staining of HK2 in HCC tissues. **b** Representative negative staining of HK2 in HCC tissues. **c** Representative positive staining of Beta2AR in HCC tissues. **d** Representative negative staining of Beta2AR in HCC tissues

**Fig. 2:**
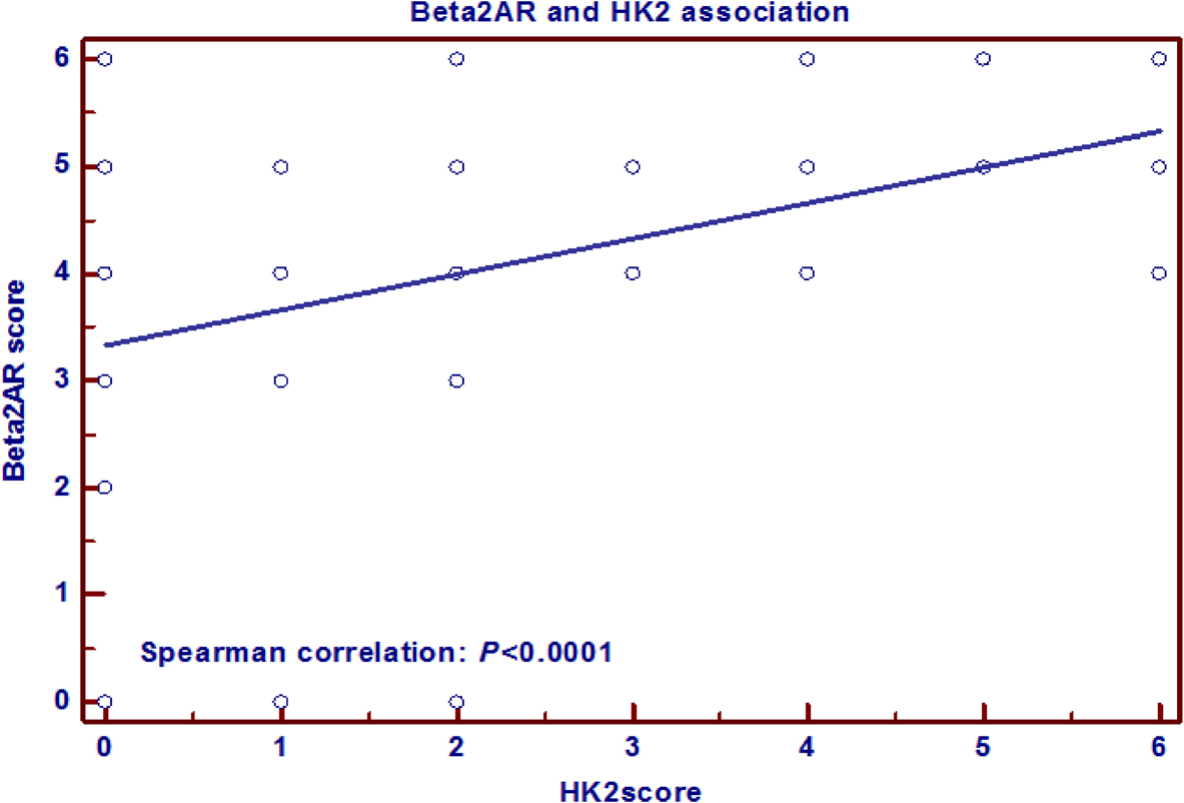
Spearman correlation between HK2 and Beta2AR. Spearman correlation test shows that HK2 and Beta2AR expression is correlated positively (*P* < 0.0001)

Positive staining of HK2 was detected in the cytoplasm of HCC tissues. And positive staining of Beta2AR was also detected in the membrane and cytoplasm of HCC tissues. Representative positive staining and negative staining of HK2 and Beta2AR in HCC tissues are shown in Fig. 1. According to the scores of staining, HK2 and Beta2AR positive expression was detected in 66 (42.58) and 122 (78.71 %) HCC samples, respectively. Spearman correlation test showed that HK2 and Beta2AR expression was correlated positively (*P* < 0.0001), as shown in Fig. 2.

### The association between HK2/Beta2AR expression and clinical data

There was no significant difference between HK2 positive staining samples and HK2 negative staining samples in age, gender, Hepatitis viral infection, AFP level, tumor number, tumor differentiation grade and post operational treatment. Only large tumor size and advanced TNM stage were correlated with HK2 positive staining positively. There was also no significant difference between Beta2AR positive staining samples and Beta2AR negative staining samples in age, gender, Hepatitis viral infection, AFP level, tumor number, TNM stage, tumor differentiation grade and post operational treatment. Only large tumor size was correlated with Beta2AR positive staining positively. The details of the association tests between HK2/Beta2AR expression and clinical data are shown in Tables 1 and 2.

**Table 1:** The details of association between Beta2AR expression and clinical data

	Beta2AR positive	Beta2AR negative	*P* value
Age			
≤ 50 year	35	7	0.391
> 50 year	87	26	
Gender			0.848
Male	98	27	
Female	24	6	
Hepatitis virus infection			0.439
HBV	106	29	
HCV	4	2	
HBV + HCV	1	1	
None	11	1	
AFP level			0.685
≤ 20ug/L	58	17	
> 20ug/L	64	16	
Tumor number			0.154
Solitary	118	30	
Multiple	4	3	
Tumor size			0.0001
≤ 5 cm	82	33	
> 5 cm	40	0	
TNM stage			0.795
I	44	12	
II	49	14	
III	22	4	
IV	7	3	
Differentiation			0.328
Well	36	14	
Moderate	58	14	
Poor	28	5	
Post operation treatment			0.483
None	77	23	
TACE	45	10

**Table 2:** The details of association between HK2 expression and clinical data

	HK2 positive	HK2 negative	*P* value
Age			
≤ 50 year	23	19	0.062
> 50 year	43	70	
Gender			0.360
Male	51	74	
Female	15	15	
Hepatitis virus infection			0.594
HBV	57	78	
HCV	3	3	
HBV + HCV	0	2	
None	6	6	
AFP level			0.054
≤ 20ug/L	26	49	
> 20ug/L	40	40	
Tumor number			0.121
Solitary	65	83	
Multiple	1	6	
Tumor size			0.001
≤ 5 cm	40	75	
> 5 cm	26	14	
TNM stage			0.015
I	26	30	
II	18	45	
III	16	10	
IV	6	4	
Differentiation			0.145
Well	19	31	
Moderate	28	44	
Poor	19	14	
Post operation treatment			0.630
None	44	56	
TACE	22	33	

### HK2 and Beta2AR can predict HCC prognosis independently and synergistically

Positive Beta2AR staining was observed in all HK2 positive HCC samples. Moreover, when Beta2AR was negative in the HCC tissues, HK2 was also negative in the corresponding samples. In the Beta2AR positive staining HCC tissues, there was no significant difference between HK2 positive samples and HK2 negative ones in age, gender, Hepatitis viral infection, AFP level, tumor number, tumor differentiation grade and post operational treatment. Only advanced TNM stage was correlated with HK2 positive staining. The details of the association tests between HK2 expression and clinical data in Beta2AR positive samples are shown in Table 3.

**Table 3:** The details of association between HK2 expression and clinical data in Beta2AR positive samples

	Beta2AR positive		
	HK2 positive	HK2 negative	*P* value
Age			
≤ 50 year	23	12	0.102
> 50 year	43	44	
Gender			0.357
Male	51	47	
Female	15	9	
Hepatitis virus infection			0.596
HBV	57	49	
HCV	3	1	
HBV + HCV	0	1	
None	6	5	
AFP level			0.050
≤ 20ug/L	26	32	
> 20ug/L	40	24	
Tumor number			0.235
Solitary	65	53	
Multiple	1	3	
Tumor size			0.091
≤ 5 cm	40	42	
> 5 cm	26	14	
TNM stage			0.006
I	26	18	
II	18	31	
III	16	6	
IV	6	1	
Differentiation			0.229
Well	19	17	
Moderate	28	30	
Poor	19	9	
Post operation treatment			0.377
None	44	33	
TACE	22	23	

**Fig. 3:**
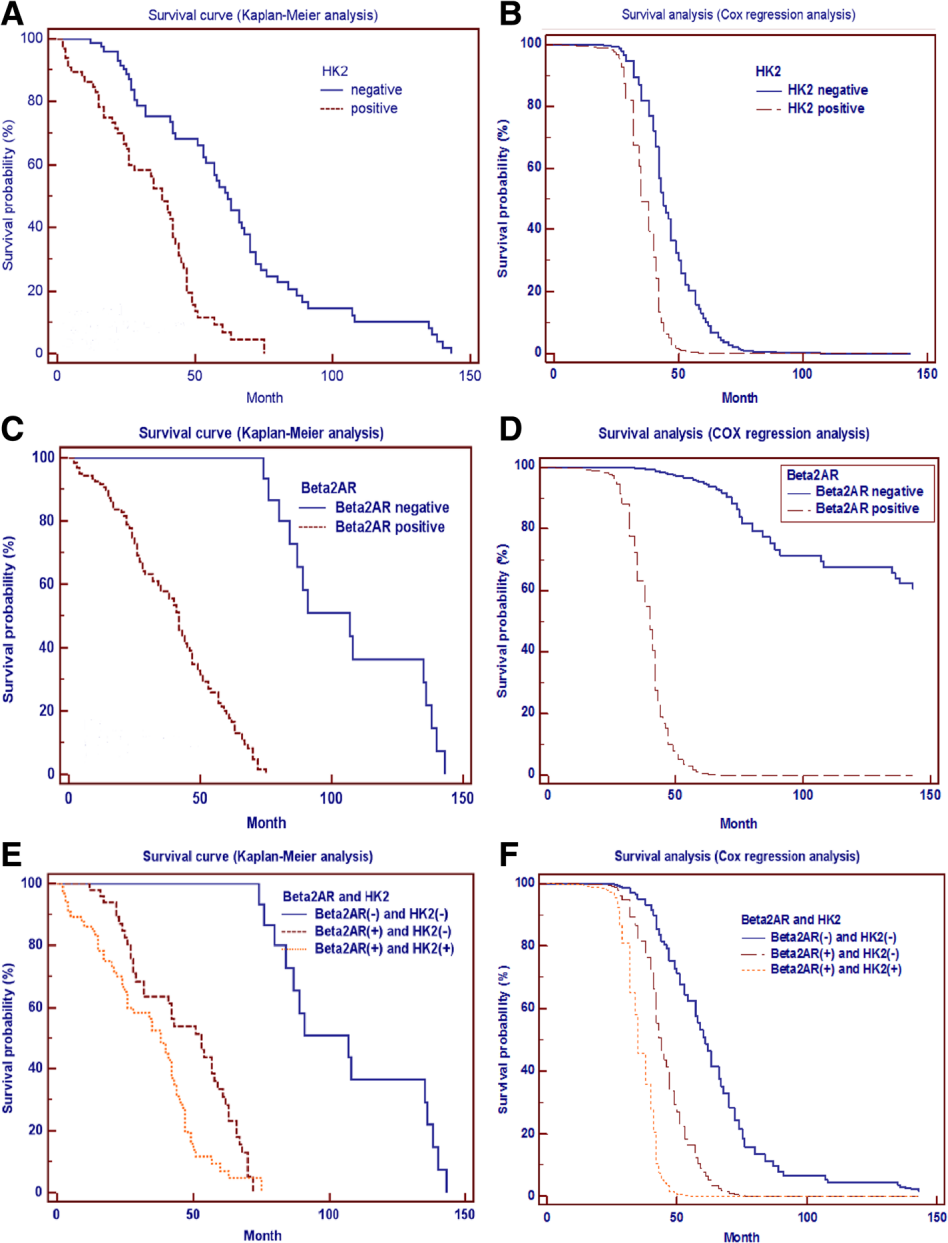
Survival curve. **a** and **b** HK2(+) is associated with poor prognosis in both univariate analysis (HR = 2.70, 95%CI = 1.76–4.15, *P* < 0.0001) and multivariate analysis (*P* < 0.0001). **c** and **d** Beta2AR(+) is associated with poor prognosis in both univariate analysis (HR = 4.61, 96%CI = 3.14–6.76, *P* < 0.0001) and multivariate analysis (*P* < 0.0001). **e** and **f** HK2(+)/Beta2AR(+) in HCC samples shows poorer prognosis as compared with HK2(−)/Beta2AR(−) in both univariate analysis (HR = 4.69, 95%CI = 2.91–7.57, *P* < 0.0001) and multivariate analysis (*P* < 0.0001). HK2(+)/Beta2AR(+) in HCC samples shows poorer prognosis as compared with HK2(−)/Beta2AR(+) in both univariate analysis (HR = 1.76, 95%CI = 1.17–2.64, *P* = 0.003) and multivariate analysis (*P* = 0.004)

In univariate analysis, HK2(+) (HR = 2.70, 95%CI = 1.76–4.15, *P* < 0.0001) and Beta2AR(+) (HR = 4.61, 96%CI = 3.14–6.76, *P* < 0.0001) were associated with poor prognosis. In multivariate analysis, HK2(+) (*P* < 0.0001) and Beta2AR(+) (*P* < 0.0001) were also associated with poor prognosis. HK2(+)/Beta2AR(+) in HCC samples had poorer prognosis as compared with HK2(−)/Beta2AR(−) in both univariate analysis (HR = 4.69, 95%CI = 2.91–7.57, *P* < 0.0001) and multivariate analysis (*P* < 0.0001). HK2(+)/Beta2AR(+) in HCC samples had the poorer prognosis as compared with HK2(−)/Beta2AR(+) in both univariate analysis (HR = 1.76, 95%CI = 1.17–2.64, *P* = 0.003) and multivariate analysis (*P* = 0.004). The survival analysis curves are shown in Fig. 3.

**Fig. 4:**
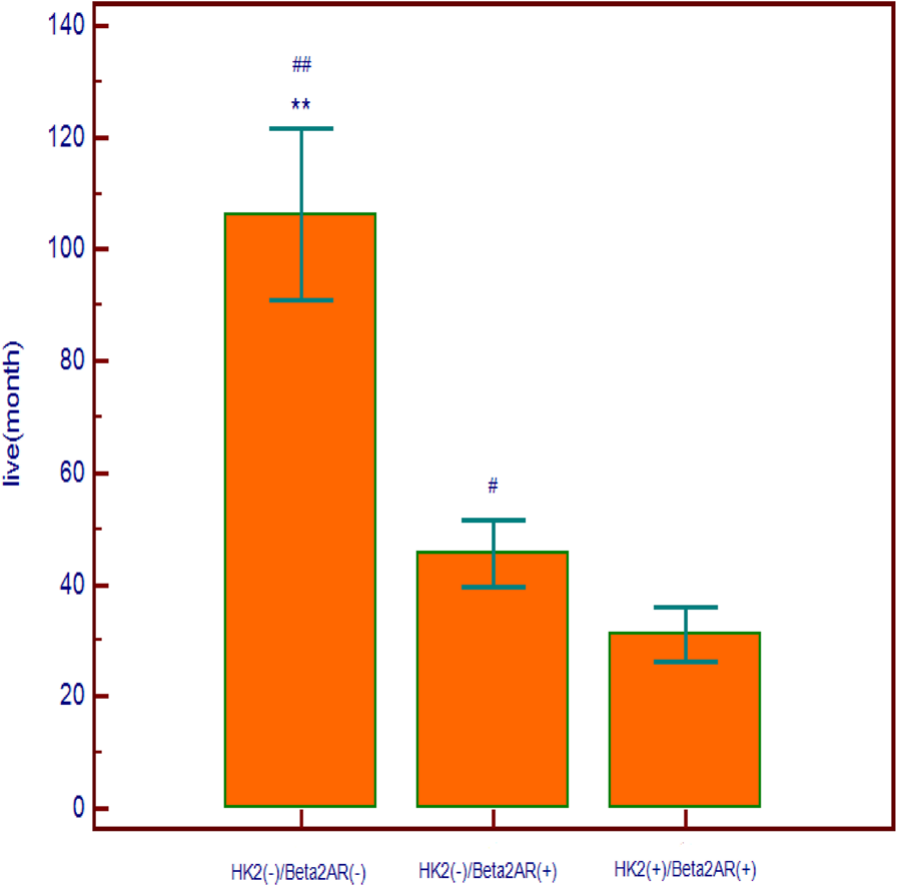
The difference of live time among different groups. One-way ANOVA analysis shows that survival time of HK2(−)/Beta2AR(−) is longer than that of HK2(−)/Beta2AR(+) and HK2(+)/Beta2AR(+) significantly. The survival time of HK2(−)/Beta2AR(+) is also longer than that of HK2(+)/Beta2AR(+). Data are shown with mean ± SD. ## *P* < 0.0001 vs. HK2(+)/Beta2(+) group, ** *P* < 0.0001 vs. HK2(−)/Beta2(+) group, #*P* = 0.001 vs. HK2(+)/Beta2AR(+) group

Regarding patients that reached death point, one-way ANOVA analysis showed that survival time of HK2(−)/Beta2AR(−) patients (106.29 ± 26.68 months) was longer than that of HK2(−)/Beta2AR(+) patients (45.61 ± 19.12 months) (*P* < 0.0001) and HK2(+)/Beta2AR(+) patients (31.13 ± 18.00 months) (*P* < 0.0001) significantly. The survival time of HK2(−)/Beta2AR(+) patients was also longer than that of HK2(+)/Beta2AR(+) patients (*P* = 0.001). The details of one-way ANOVA analysis are shown in Fig. 4.

## Discussion

HCC is the third most common cause of death from carcinomas worldwide [1, 2]. Moreover HCC is also one of the main causes of death of liver cirrhosis irrespective of cirrhotic etiology [19]. Due to the increasing incidence of hepatitis C virus (HCV) infections and non-alcoholic fatty liver disease (NAFLD), the incidence of HCC in USA has doubled in the past twenty years. Based on the trend of HCC incidence, it is postulated that HCC is likely to replace breast cancer and colorectal cancer to become the third cause of death from carcinomas in USA [20]. Different from developed countries, the etiology of HCC in developing countries especially southeastern Asian countries is hepatitis B virus (HBV) infection [21, 22]. The high incidence of HBV infection also makes HCC to be a big healthy problem in southeastern Asian countries. Irrespective of the etiology of HCC, the prognosis of HCC mainly relies on diagnosis at early stage and the possibility of curative surgical resections. However, even in USA, the rate of HCC diagnosed at early stage is only 46 % [20]. And only a small portion of HCC patients diagnosed at early stage can receive curative resections [20]. In the developing countries, the rates of HCC diagnosed at early stage and the portions of HCC patients at early stage received curative resections are very low. Although tyrosine kinase inhibitors (such as Sorafeniband Linifanib) have been developed to treat HCC at advanced stage, the outcome of treatment at advanced stage is not satisfactory [3, 4]. Scientists are still searching for new HCC treatment targets. Exploring the mechanism of HCC development may shed light on developing new therapies. Another realistic issue of importance to clinicians and patients is to predict prognosis of HCC. Proper prediction of prognosis can guide clinicians to choose the right therapies and help patients to arrange the remaining life appropriately.

Beta2AR regulates cell proliferation via cyclic adenosine monophosphate and protein kinase A pathways [5]. Through increasing DNA synthesis, accelerating cell cycle and decreasing apoptosis, activation of Beta2AR promotes cancer growth, invasion and metastasis. A previous study indicates that high expression of Beta2AR in HCC tissues is a biomarker for poor prognosis [10]. The Kaplan-Meier survival analysis and Cox regression analysis of our study are in consistence with the findings of this study. Our study also proves that high expression of Beta2AR in HCC is associated with large tumor size, which implies high expression Beta2AR in HCC playing an important role in tumor growth.

Glycolysis is the the key step for cellular energy metabolism, which converts glucose into pyruvate to produce adenosine triphosphate (ATP) [23]. Ten steps and corresponding enzymes participate in this process. Among these enzymes, HK is the first step enzyme converting glucose into glucose 6-phosphate, and pyruvate kinase (PK) is the final step enzyme converting phosphoenolpyruvate into pyruvate [23]. When abundant oxygen is present, pyruvate enters tricarboxylic acid (TCA) cycle to produce ATP in mitochondria. Under hypoxia conditions, pyruvate will be converted into lactate by lactate dehydrogenase (LDH) to produce ATP. Tumor prefers the latter instead of entering the TCA cycle to produce energy, even under sufficient oxygen conditions, which is called Warburg effect [11]. HK and PK play important roles in this energy shift [24]. To date, four HK isoforms and four PK isoforms are identified in mammals [12, 25, 26]. Different from normal liver cells, HCC tissues express HK2 and PKM2 predominantly [13, 14, 27]. This isoform alteration makes cancer cells prefer aerobic glycolysis instead of aerobic oxidation [11]. Accelerated aerobic glycolysis supplies abundant energy to cancer cells for their survival, growth and metastasis [11]. HK2 participates in tumor initiation and maintenance, and HK2 depletion inhibits the neoplastic phenotype of human lung and breast cancer cells in vitro and in vivo [25]. Previous study showed that 3-bromopyruvate (3-BP), a HK2 inhibitor induced apoptosis of HCC cells via augmenting ER stress and anti-angiogenesis by protein disulfide isomerase inhibition [28]. In another study, Resveratrol (a HK2 inhibitor) also induces apoptosis of HCC cells via inhibiting aerobic glycolysis [29]. All the above evidence indicates that HK2 might be a potential therapeutic target for HCC.

Recently one study also revealed that the levels of HK2 expression in dysplastic cirrhosis and HCC was higher than that of non dysplastic cirrhosis and normal liver, which indicates that high expression of HK2 is associated with high grade of malignancy [30]. Previous studies also showed that high expression of HK2 was an independent poor prognostic biomarker for HCC [13, 14]. The results of our study are in consistence with these two survival studies. Our study also suggests that high expression of HK2 is associated with large tumor size and advanced TNM stages, which indicates that high expression of HK2 in HCC represents high levels of malignancy. High HK2 expression in HCC tissues can also be used as a biomarker for poor HCC prognosis.

Moreover, HK2 and Beta2AR are correlated in HCC development. Previous study indicates that activation of Beta2AR can promote HK2 expression, and inhibition of Beta2AR can decrease HK2 expressions in breast cancer cell lines [15]. Our study also shows positive associations between HK2 and Beta2AR in HCC tissues. Additionally, Beta2AR positive staining in HCC is not associated with TNM stage in our study. However, in Beta2AR positive stained samples, HK2 positive staining is associated with TNM stage. This may imply that Beta2AR increases HCC invasion and metastasis through HK2 activation. Detecting HK2 and Beta2AR simultaneously may help clinicians to evaluate the malignant status of HCC. Combination therapy of targeting HK2 and Beta2AR might be a promising strategy for HCC.

Combination of HK2 and Beta2AR detection also improves the predictive power for prognosis. The Kaplan-Meier survival analysis and Cox regression analysis of our study showed that HK2(+)/Beta2AR(+) HCC patients had poorer prognosis compared with HK2(−)/Beta2AR(+) and HK2(−)/Beta2AR(−) HCC patients. Detecting HK2 and Beta2AR expression in HCC simultaneously has clinical significance in prognosis prediction. However, there are some limitations of our study. Firstly, the sample number of our study is not very large, which could attenuate the statistical power. Secondly, because of the limitations of pathologic samples, only immunohistochemistry was employed in our study, other methods to detect HK2 and Beta2AR (Western blot and quantitative real time PCR) are needed to confirm the results of this study. Moreover, in vitro studies are also required to prove these findings. Thirdly, we were not able to calculate the score of Child Pugh due to incomplete information collection, the effect of liver function reservoir on HCC survival and Cox regression analyses could not be evaluated. Fourthly, we were unable to retrieved the recurrence information of HCC, so we did not evaluate the expression of HK2 and Beta2AR on the recurrence of HCC. Despite of the above limitations, our study will shed new light on HCC treatment and prognosis predictions.

## Conclusion

In summary, Beta2AR and HK2 expression in HCC tissues is positively correlated. High expression of HK2 and beta2AR in HCC can predict prognosis of HCC patients independently and synergistically. Beta2AR might increase the grade of malignancy of HCC via promoting HK2 expression. Combined targeting of HK2 and Beta2AR might be a promising therapy for HCC.

## Abbreviations

BCLC, Barcelona Clinic Liver Cancer algorithm; Beta2AR, Beta2-adrenergic receptor; CT, computed tomography scanning; GPCR, transmembrane G protein-coupled receptor; HBV, hepatitis B virus; HCC, hepatocellular carcinoma; HCV, hepatitis C virus; HK2, Hexokinase-2; MRI, magnetic resonance images.
